# Effects of intense storm events on dolphin occurrence and foraging behavior

**DOI:** 10.1038/s41598-020-76077-3

**Published:** 2020-11-06

**Authors:** Amber D. Fandel, A. Garrod, A. L. Hoover, J. E. Wingfield, V. Lyubchich, D. H. Secor, K. B. Hodge, A. N. Rice, H. Bailey

**Affiliations:** 1 Chesapeake Biological Laboratory, University of Maryland Center for Environmental Science, Solomons, MD USA; 2 Center for Conservation Bioacoustics, Cornell Lab of Ornithology, Cornell University, Ithaca, USA

**Keywords:** Ecology, Behavioural ecology, Ecosystem ecology

## Abstract

As storms become increasingly intense and frequent due to climate change, we must better understand how they alter environmental conditions and impact species. However, storms are ephemeral and provide logistical challenges that prevent visual surveys commonly used to understand marine mammal ecology. Thus, relatively little is known about top predators’ responses to such environmental disturbances. In this study, we utilized passive acoustic monitoring to characterize the response of bottlenose dolphins to intense storms offshore Maryland, USA between 2015 and 2017. During and following four autumnal storms, dolphins were detected less frequently and for shorter periods of time. However, dolphins spent a significantly higher percentage of their encounters feeding after the storm than they did before or during. This change in foraging may have resulted from altered distributions and behavior of their prey species, which are prone to responding to environmental changes, such as varied sea surface temperatures caused by storms. It is increasingly vital to determine how these intense storms alter oceanography, prey movements, and the behavior of top predators.

## Introduction

Coastal shelf marine ecosystems are both highly productive and particularly susceptible to disturbances through natural events and anthropogenic activities. Disturbances, such as storm events, often alter sound or nutrient levels in the environment^1^, increase turbulent mixing of the water column, and rapidly change ocean salinity and temperature gradients^2,3^. Such environmental changes can affect the distribution and behavior of a diverse array of shelf fauna^4,5^ and have long-lasting effects on inshore ecosystems such as coral reefs^6^ and other hard-bottom habitats. Extreme storm events are expected to become more frequent^7,8^ and intense^8–10^ as human-driven climate change continues to alter oceanic conditions. Determining how apex predators respond to storms is an important component in understanding how climate change mediates top-down effects in marine ecosystems.

Opportunistically recorded changes in several marine species’ behavior and distribution have been attributed to storms. Passive acoustic monitoring revealed that spotted seatrout^11^ (*Cynoscion nebulosus*) and sand seatrout^4^ (*Cynoscion arenarius*) altered their vocalizations and shifted the timing of their spawning activity both during and immediately after hurricanes. Temporary and permanent range shifts in several shark^12^ and fish species^13,14^ have also been observed following intense storm activity, although the response varied in duration and amongst species and age classes. More extreme impacts have included increased mortality or decreased abundance in macrobenthic organisms because of changes in oxygen concentrations^15^ and in juvenile fish^13^ and sessile invertebrates^16^ as a result of wave action. The environmental drivers of many of these organismal responses, however, are not fully understood.

As top predators, marine mammals may be directly affected by bottom-up changes, environmental changes caused by storm events or indirectly through changes in their prey. Storm events were considered the direct cause of bottlenose dolphin (*Tursiops truncatus*) movement into shallower waters^17,18^ and lower adult survival probabilities for manatees (*Trichechus manatus latirostris*) in years with intense hurricanes^19^. Following two hurricanes, Bahamian dolphin populations experienced 30% population loss, but it is not known whether this was due to emigration and/or mortality^20^. Hurricanes in the Mississippi Sound area had variable effects on short^21^ and long-term dolphin foraging activities^22^. In addition to altering salinity and temperature gradients, storms can alter upwelling patterns and lead to harmful algal blooms^23,24^ including lethal marine mammal domoic acid poisoning^25^. Increased sound levels from anthropogenic sources caused short-term changes in odontocete foraging^26–28^ and communication^28–30^. While we increasingly understand how anthropogenic noise impacts marine mammals, it is not yet clear how elevated sound levels from natural sources, such as those caused by high wind and wave action during storms, affects their behavior and ecology.

**Figure 1:**
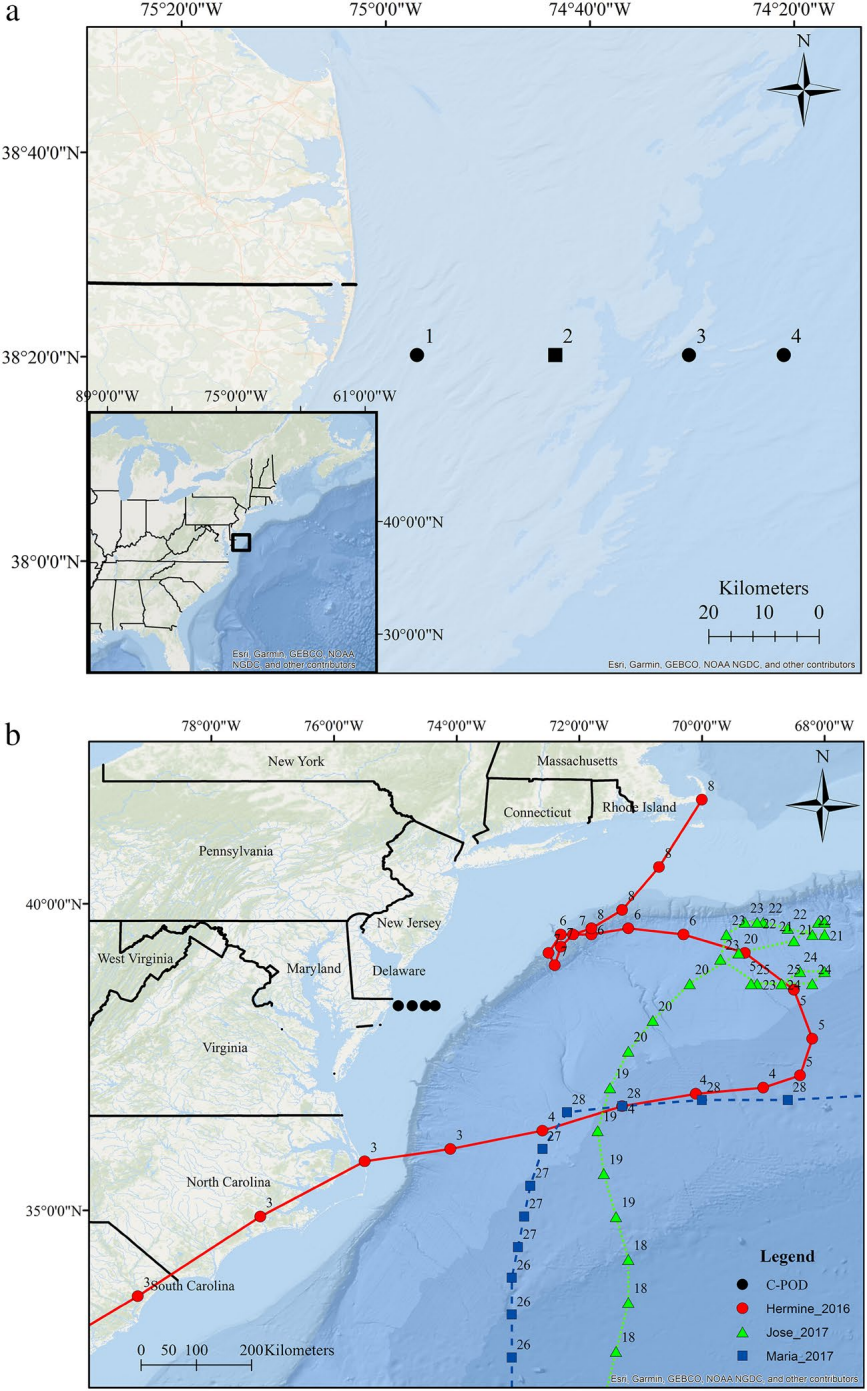
Map of the Mid-Atlantic coast of the United States with the (**a**) four C-POD deployment sites (black dots) and Site 2, at which the MARU and SM3M were also deployed (black square), and (**b**) maps of C-POD sites and locations of the center of the tropical storms during September of 2016 (Hermine: red, red circle) and 2017 (Jose: green, green triangle and Maria: blue, blue square). Labels on the storm tracks indicate the date (in September) of the storm data (data from the US National Oceanic and Atmospheric Administration’s (NOAA’s) Tropical Cyclone Reports; www.nhc.noaa.gov/data/#tcr). The map was constructed in Esri ArcGIS 10.4 (www.esri.com) using state and oceanographic features from Esri ArcGIS Online (www.esri.com/en-us/store/arcgis-online).

In this study, we analyzed passive acoustic detections of bottlenose dolphin calls within the US Mid-Atlantic Bight to determine whether dolphin occurrence and foraging behavior were affected by intense storm events (Table 1, Fig. 1b). Passive acoustic monitoring is advantageous to traditional visual monitoring techniques, especially during storm events, because it does not require favorable viewing conditions for marine mammal detection and dramatically extends the observation period. The US Mid-Atlantic Bight is a large shelf region in the Northwest Atlantic Ocean spanning subtropical and temperate zones that are susceptible to rapid changes in ocean conditions caused by autumnal storm events. During these intense storm events, flow conditions change, environmental noise is increased, and shelf waters rapidly destratify causing large temperature changes^14,31^. Common dolphins (*Delphinus delphis*), spotted dolphins (*Stenella frontalis*), and bottlenose dolphins are present in this region^32^. During the summer and fall, bottlenose dolphins are the primary species sighted^32–35^, and they feed on a variety of fish and squid species^36,37^. While bottlenose dolphins and other marine mammals are highly mobile, adaptable, and potentially less directly impacted by changes in water temperature than ectothermic species, they could be indirectly affected by alterations in prey behavior and distribution. We hypothesized that dolphin detections and foraging behavior in the Mid-Atlantic Bight would consequently be reduced during and following intense storm events such as tropical storms due to altered prey distribution^14^ and avoidance of the storm. In the future, marine ecosystems may be increasingly exposed to disturbances such as storm events, which are expected to occur with higher frequency and intensity as the climate changes^8^.

**Table 1:** Year, name of storm, days analyzed before and after the storm period, arrival and departure dates of the storm, and for the days during each storm, the maximum wind speeds and the dates on which they occurred, mean wind speeds with standard deviation, minimum wind speed, and maximum wind gust speeds.

Year	Name	Before	During						After
		Dates	Dates	Maximum wind speed date	Maximum wind speed (m/s)	Mean wind speed (m/s ± SD)	Minimum wind speed (m/s)	Maximum wind gust speed (m/s)	Dates
2015	Unnamed extratropical storm	16 Aug–30 Sep (46 days)	1–4 Oct (4 days)	3 Oct	13.5	7.0 ± 2.2	1.9	22.2	5–15 Oct (11 days)
2016	Tropical Storm Hermine	16 Aug–2 Sept (18 days)	3–8 Sep (6 days)	3 Sep	11.4	5.1 ± 2.1	0.0	18.7	9 Sept–15 Oct (37 days)
2017	Tropical Storm Jose	16 Aug–18 Sept (34 days)	19–23 Sep (5 days)	19 Sep	12.3	4.6 ± 2.2	0.0	18.8	N/A
	Tropical Storm Maria	N/A	27–28 Sep (2 days)	28 Sep	10.7	6.3 ± 1.6	2.1	14.6	29 Sept–15 Oct (17 days)

## Results

**Figure 2:**
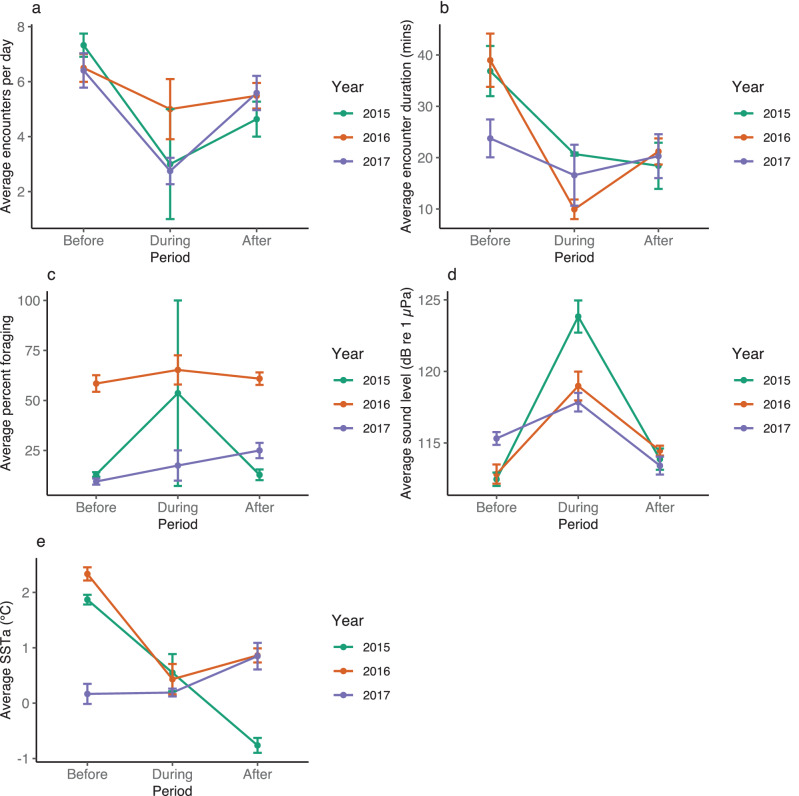
Average values (mean ± SE) for 2015 to 2017 in the before, during, and after storm periods for (**a**) encounters per day, (**b**) daily encounter length (min), (**c**) daily percent of encounter spent foraging, (**d**) daily sound level (dB re 1 µPa rms), (**e**) daily sea surface temperature anomalies (SSTa; °C).

Here we present results based on the mixed-effect estimates, ${\widehat{b}}_{j}$ (j = 0, 1, 2), from model (1) for each metric. There were significantly fewer daily dolphin encounters during (mean = 2.80, n = 17, p < 0.01) and after (mean = 5.26, n = 65, p = 0.02) storms compared to before storms (mean = 6.77, n = 97; Table 2, Fig. 2a). When dolphins did occur during and following storms, encounters were significantly shorter than they were before the storms [mean = 2.55 log(min), n = 11, p = 0.04 ; mean = 2.76  log(min), n = 65, p = 0.02, respectively; Table 2, Fig. 2b]. However, the percentage of time dolphins spent foraging during an encounter increased after the storms (mean = 32.75, n = 64, p = 0.02; Table 2, Fig. 2c). Ambient sound levels also increased significantly during storms (mean = 119.74 SPL rms, n = 17, p < 0.01; Table 2, Fig. 2d), and how the storm affected SSTa varied (Fig. 2e).

**Table 2:** Results of the mixed-effects models for the dolphin encounter, behavior, and environmental metrics, indicating the effects of the storm (relative to the period before storm) on each.

Metric	Period	Estimate, $\widehat{b}$	Std. error	t	p-value	Effects
Number of encounters	Before	6.77	0.42	16.09		Year, Auto-correlation
	During	− 3.97	0.99	− 4.02	< 0.01*	
	After	− 1.51	0.66	− 2.28	0.02*	
Encounter duration (log (min))	Before	3.16	0.12	26.79		Year, Auto-correlation
	During	− 0.61	0.29	− 2.11	0.04*	
	After	− 0.40	0.17	− 2.33	0.02*	
Percent foraging	Before	26.75	15.04	1.78		Year, Heterogeneous variance
	During	13.74	8.57	1.60	0.11	
	After	6.00	2.60	2.31	0.02*	
Sound level (SPL rms)	Before	113.43	0.42	267.25		Year, Auto-correlation
	During	6.31	0.99	6.40	0.00*	
	After	0.73	0.66	1.10	0.27	
SSTa (°C)	Before	1.29	0.44	2.90		Year, Auto-correlation
	During	− 0.41	0.22	− 1.88	0.06	
	After	− 0.62	0.46	− 1.35	0.18	

The effects (heterogeneous variance and/or auto-correlation) included in the final model were chosen using the AIC values for each model. Year was included as a random effect in every model to account for inter-annual variation. An asterisk indicates statistical significance (p-value < 0.05).

For all models, ANOVA F-tests confirmed that the means of each variable were statistically different by period (the p-values for the individual models were adjusted for the number of tests using the Benjamini–Hochberg procedure^38^). Mean values were obtained from dolphin occurrence and behavior metrics across periods, with p-values accounting for the random or autocorrelation effects structure in the final models (Table 2). Residual diagnostics plots were used to check homogeneity, possible covariance, and normality of model residuals. One of the models (for encounter duration) required a logarithmic transformation of the response to achieve normality of the residuals; however, both the transformed and untransformed models indicated the high statistical significance of the storm effects.

For the 4 days in 2017 on which the C-POD detected dolphins but did not indicate foraging, concurrent sound files from the SM3M acoustic recorder were examined to verify that no detectable foraging occurred. The C-POD, as a conservative classifier of dolphin clicks, missed two feeding buzzes. On the loudest days in 2016 and 2017, dolphin presence and foraging were detected despite elevated ambient sound levels during the storms.

## Discussion

Our study found that storms in the US Mid-Atlantic Bight caused sea surface temperature anomalies to vary, as was expected based on the mixing of the stratified water column with cooler bottom water, thus lowering the surface temperature, as found in other studies^14^. While lower water temperatures are unlikely to have directly affected the dolphins’ behavior, their ectothermic prey species are influenced by these changes^14,39^. The resulting changes in prey distribution may have indirectly influenced dolphin movements and foraging. The significantly lower dolphin occurrence and shorter encounters indicate that dolphins had to search more widely for prey during the storms or alter their foraging strategies, but were able to increase the percentage of time spent foraging after the storms compared to before storms (Table 2, Fig. 2c). They could also be compensating for reduced foraging during the storm, taking advantage of the changed behavior and disorientation of fish following the disturbance and changed conditions caused by the storm.

Bottlenose dolphins are highly mobile animals that can have large ranges of 500 km or more^40^ and are generalist carnivores^36,37^. Their diet is composed of cephalopods, crustaceans, and large and small fish species^36,37,41^. The movements and concentrations of their prey are influenced by a variety of environmental factors including water temperature^14^, salinity^15^, oxygen concentration^15,42^, barometric pressure, and wind speeds^12^, all of which are altered by storms. In California, bottlenose dolphins extended their range in response to prey shifts during El Niño^43^. Similar short-term changes in dolphin presence, as assumed from fewer recorded dolphin vocalizations, were also shown in our study. However, this reduction in recorded vocalizations may have also resulted from changes in group size or vocal behavior. Further research on the foraging techniques, prey choices, and specific prey responses to storms would improve our understanding of the cause of top predators’ responses to these disturbances.

Fish species exhibit highly variable responses to storms. While environmental changes elicited permanent range shifts in some prey species^14^, short-term distributional changes (on the scale of days) were found in others^12,44^. Vertical and horizontal range shifts in fish to avoid hypoxic zones caused by storm events have also been observed^41^. During Tropical Storms Jose and Maria, intense wave action caused gray triggerfish (*Balistes capriscus*) in North Carolina to increase their emigration rates^44^. Concurrent research in our study region found that black sea bass (*Centropristis striata*) evacuated the region as a result of Tropical Storm Hermine in 2016^14^. Water column mixing and changing bottom temperatures initiated a migration of 40% of tagged black sea bass and caused reduced activity rates by those that remained^14^. When environmental changes, including those from storm events, result in fish that are stunned, less active, or concentrated in a region, it potentially increases their catchability by predators. This prey behavior is a possible cause of increased dolphin foraging following storm events. After two intense hurricanes in Mississippi, bottlenose dolphin foraging increased 15% over pre-hurricane levels and remained heightened for 2 years, potentially due to increased biodiversity, decreased vessel traffic, or less fishing as a result of the storm^22^.

Although storms may create unique foraging opportunities for dolphins, severe wave action from storms has displaced dolphins^17,18,45^ and caused natural ambient sound levels to peak during storms. Exposure to elevated sound levels from anthropogenic sources can result in abandonment of important habitat^46^ or a diminished ability to compensate for short-term changes in activities such as feeding^47^. Should dolphins experience range shifts due to direct displacement or in search of prey, they may be exposed to changes in pollutants, pathogens^45^, and predators^48^. Interspecific dynamics^20^ and biodiversity have also been affected by range shifts in these top predators^48^. Bottlenose dolphin and spotted dolphin (*Stenella frontalis)* populations in the Bahamas decreased in size by 30% following two hurricanes, likely due to death and/or emigration^20^. These dramatic effects are increasingly likely as dolphins are forced to cope with the cumulative stress of altered prey distributions, physical and acoustic disturbances, and changing environmental conditions^49^.

While dolphins were detected throughout our study period, including on days with high ambient sound levels (up to 123 dB re 1 µPa rms), elevated ambient sound levels during the storms likely masked dolphin clicks^50,51^ or decreased the detection range of the C-POD^52^. The C-POD’s KERNO classifier is also a conservative classifier of dolphin clicks^53^. While no dolphins were detected during the 2 loudest days of our study period (average sound levels of up to 126 dB re 1 µPa rms in 2015), on the loudest days in 2016 and 2017, dolphin presence and foraging were detected despite elevated ambient sound levels during the storm. A previous study in this region showed that the C-POD’s ability to detect dolphins (based on click trains between 20 and 160 kHz) was not significantly affected by broadband (2 Hz–24 kHz, up to 130 dB re 1 µPa rms) ambient sound levels^53^. Storm noise, which has most of its energy in lower frequencies, did not appear to greatly reduce detections by the C-POD. Our review of concurrent sound files from the SM3M acoustic recorder similarly indicated decreased occurrence of feeding buzzes rather than masking by the storm noise. The C-POD’s passive acoustic click data allowed us to measure the number of dolphin encounters per day, but we were unable to determine the number or identity of dolphins in the area at any time. As a result, we could not measure the effects of storms on dolphin abundance. Dolphin signature whistles recorded with devices like the SM3M, however, allow identification of individuals^54^ and could offer future insight into how many dolphins were impacted by storm events and whether they permanently evacuated or returned.

Understanding the effects of storms on top predators such as marine mammals can serve as an indicator for the overall resiliency of the ecosystem they inhabit. Yet the key to understanding trophic level responses is rooted in understanding the changes in environmental conditions. As storms become increasingly frequent and intense due to climate change, it is vital to determine how storms alter oceanography and prey movements. This will provide a more complete understanding of storms as drivers of behavioral changes in dolphins and other top predators.

## Methods

### Study area

Our study area was located in the US Mid-Atlantic Bight between 12 and 63 km east of Ocean City, Maryland, USA (Fig. 1a). At the four sites in this area (Site 1: nearest inshore—Site 4: farthest offshore, Fig. 1a), water depths ranged from approximately 20–45 m, and acoustic recording instruments were deployed with the hydrophone located approximately 1.5 m above the ocean floor using bottom-anchored moorings (see^53,55^ for mooring configurations). Automatic dolphin click detectors (C-PODs; Chelonia Ltd., UK) were deployed at all four sites from 2015 through 2017, a Marine Autonomous Recording Unit (MARU; Cornell Lab of Ornithology Bioacoustics Research Program, Ithaca, NY) continuously monitored sound levels at a sampling rate of 2 kHz [15 Hz–1 kHz] at Site 2 from 2015 through 2017, and an SM3M acoustic recorder (Wildlife Acoustics, Maynard, MA, USA) sampling at 48 kHz [2 Hz–24 kHz] was deployed at Site 2 from May of 2016 through 2017 to verify dolphin detections (Fig. 1a).

### Storm and environmental data

One strong extratropical storm (2015) and three classified tropical storms (2016 and 2017) that occurred during the late summer and autumn near the study area were tracked using data obtained from the U.S. National Oceanic and Atmospheric Administration’s (NOAA’s) Tropical Cyclone Reports (www.nhc.noaa.gov/data/#tcr) and wind speeds were obtained from NOAA’s National Buoy Data Center (www.ndbc.noaa.gov; Station OCIM2; Table 1). Late summer and autumn storms (16 August–15 October) were chosen for analysis because dolphins are most common in this coastal region during that period^33,34,56^. In 2015, a strong storm event took place within the study area on 1–4 October with wind speeds up to 13 m/s and gusts up to 22 m/s (Table 1). In 2016, Tropical Storm Hermine passed through the study area on 3–8 September with wind speeds up to 11 m/s and gusts up to 19 m/s (Fig. 1b; Table 1). In 2017, Tropical Storms Jose and Maria moved through the region on 19–23 and 27–28 September, respectively, with wind speeds up to 12 m/s and 11 m/s and gusts up to 19 m/s and 15 m/s, respectively (Fig. 1b; Table 1). The days from 16 August to the date of storm arrival were categorized as the period before the storms (Table 1). The days on which the storms’ winds affected conditions in the study area (1–4 October 2015, 3–8 September 2016, and 19–23 and 27–28 September 2017) were categorized as the period during the storms, and the days since the storms’ departure to 15 October were categorized as the periods after the storms. The period of 24–26 September 2017 was omitted to ensure the before and after periods were at least 10 days long (Table 1).

Sea surface temperature anomaly data (°C, SSTa) were downloaded from NOAA’s ERDDAP database^57^. These data were provided by the Group for High Resolution Sea Surface Temperature (GHRSST), and SSTa were calculated as a retrospective dataset, comparing daily temperatures with a 1 km spatial resolution to historically averaged climatology between 2003 and 2014^58^.

### Acoustic data analysis

#### Dolphin detections

C-PODs were utilized to determine the occurrence of bottlenose dolphins*.* C-PODs continuously monitor for dolphin click trains (five or more clicks) between 20 and 160 kHz, detecting dolphins up to 1.8 km away^52^. Only click trains classified as being of high and moderate qualities by the C-POD’s KERNO cetacean classifier were included in the analysis. C-PODs were deployed at each of the four sites during the 2015 through 2017 study period (Fig. 1a). To determine whether storms affected the C-POD’s dolphin detection ability, an SM3M acoustic recorder sampling at 48 kHz ([2 Hz–24 kHz]; hydrophone sensitivity: 165 dB re 1 V/µPa, gain 12 dB re 1 µPa) was also deployed on the mooring at Site 2 in 2016. Spectrograms from the SM3M recordings were manually reviewed in Raven Pro 1.5 (Cornell Lab of Ornithology Bioacoustics Research Program, Ithaca, NY) to determine whether dolphin foraging occurred during storm events in 2016 and 2017. The SM3M was not yet gathering data when the 2015 storm occurred.

#### Dolphin occurrence and behavior metrics

To determine how dolphin behaviors changed as a result of storms, behavior metrics were calculated from the C-POD data. These metrics were the total number of encounters per day, average daily encounter length, and daily average proportion of minutes per encounter spent foraging. Because C-PODs are conservative indicators of dolphin presence^53^, an encounter was defined as a sequence of detections with a new encounter being assigned when there were more than 37 min between detections (as described in^56^). Foraging behavior was defined as the occurrence of an inter-click interval (ICI) of 9.9 ms or less^56^. Any minute in which this short ICI, a feeding buzz^26,56,59^, occurred was labeled a foraging minute. Feeding buzzes are associated with the majority of prey interceptions in bottlenose dolphins^60^. The percentage of minutes spent foraging during an encounter was calculated for each encounter and these percentages were averaged at the daily scale for analysis.

#### Ambient sound levels

Underwater sounds were analyzed from the MARU, which recorded continuously at a 2 kHz sampling rate [15 Hz–1 kHz]. Because sampling was performed continuously, a very low sampling rate was selected to maximize battery life. While dolphins do not echolocate in such low frequencies, they can hear within this range^61^, and much of the ambient sound energy is within this bandwidth^62^. The MARU was anchored in the vicinity of the C-POD at Site 2. Acoustic data were processed using the Raven-X toolbox^63^ in MATLAB (Mathworks, Inc.). The sound pressure level was used to calculate the root-mean-square (rms) pressure within one-hour time bins to represent the ambient sound levels. These binned values were averaged to obtain the mean daily ambient sound levels. As the relationship between ocean ambient sound levels and wind speed, especially during storms, has been well established^64,65^, sound level was used as an indication of storm intensity.

### Statistical analysis

Linear mixed-effects models^66^ were used to determine whether any change in dolphin behavior was associated with storms. The model for each metric included all 3 years, and the year was treated as a random effect to account for inter-annual differences. The models determined the effect of the period (before, during, and after the storm) on each environmental (sound level and SSTa) and dolphin (number of dolphin encounters, encounter length, percentage of minutes per encounter spent foraging) metric. The linear mixed-effects model took the form:

1 $$Y_{i} = b_{0} + b_{j} + \alpha_{Year} + \varepsilon_{ij} ,$$

where *Y*_*i*_ is the dolphin encounter or environmental metric, *i* is the date of the observation, *b*_0_ is the grand intercept or the reference level during the period before the storm, *b*_*j*_ is the effect of storm period relative to the period before the storm (*j* = 1, 2 for during and after the storm), α_Year_ is the random intercept varying by year to account for differences in mean responses simply due to the use of different years of data. The residuals ε_*ij*_ were assumed to be uncorrelated, with a mean of 0 and variance σ^2^ (Var(ε_*ij*_) = σ^2^), or with heterogeneous variance by period (Var(ε_*ij*_) = ${\sigma }_{j}^{2}$), or autocorrelated and following an autoregressive process of first order, AR(1) (Cov(ε_*ij*_, ε_*i*−1,*j*_) = φσ^2^, where |φ|< 1 is the autoregressive coefficient).

Models were fit in the statistical software R (Version 3.5.2; R Core Team, 2018) using the package lme4^67^. The final model for each response metric was chosen based on the smallest value of Akaike information criterion (AIC) from the alternative model specifications (presence or absence of the random intercept and different structure of the residual covariance).
